# Meta-analysis of sex differences in gene expression in schizophrenia

**DOI:** 10.1186/s12918-015-0250-3

**Published:** 2016-01-11

**Authors:** Wenyi Qin, Cong Liu, Monsheel Sodhi, Hui Lu

**Affiliations:** Department of Bioengineering, University of Illinois at Chicago, 851 S. Morgan, Rm 218, Chicago, IL 60607 USA; Department of Pharmacy Practice and Center for Pharmaceutical Biotechnology, University of Illinois at Chicago, 900 S Ashland Ave mc870, Chicago, IL 60607 USA; SJTU-Yale Joint Center for Biostatistics, Shanghai Jiaotong University, Shanghai, China

**Keywords:** Dorsolateral prefrontal cortex, Microarray analysis, Postmortem brain

## Abstract

Schizophrenia is a severe psychiatric disorder which influences around 1 % of the worldwide population. Differences between male and female patients with schizophrenia have been noted. There is an earlier age of onset in males compared with females with this diagnosis, and in addition, there are differences in symptom profiles between the sexes. The underlying molecular mechanism of sex difference remains unclear. Here we present a comprehensive analysis to reveal the sex differences in gene expression in schizophrenia with stringent statistics criteria. We compiled a data set consisting of 89 male controls, 90 male schizophrenia patients, 35 female controls and 32 female schizophrenia patients from six independent studies of the prefrontal cortex (PFC) in postmortem brain. When we tested for a sex by diagnosis interaction on gene expression, 23 genes were up-regulated and 23 genes were down-regulated in the male group (q-value < 0.05), several genes are related to energy metabolism, while 4 genes are located on sex chromosome. No genes were statistically significant in the female group when multiple testing correction were conducted (q-value <0.05), most likely due to the small sample size. Our protocol and results from the male group provide a starting point for identifying the underlying different mechanism between male and female schizophrenia patients.

**Electronic supplementary material:**

The online version of this article (doi:10.1186/s12918-015-0250-3) contains supplementary material, which is available to authorized users.

## Introduction

Schizophrenia is a severe psychiatric disorder with a population frequency of approximately 1 % [30]. Schizophrenia is a syndrome characterized by positive symptoms such as delusions, hallucinations, disorganized speech and grossly disorganized or catatonic behavior; negative symptoms such as affective flatterning, alogia, or avolition [26, 30]. The etiology and pathophysiological mechanisms of the disorder are not well understood. Research to date indicates that schizophrenia is a multi-factorial neurodevelopmental impairment of the brain that could be attributed to both genetic and environmental factors [2, 7, 21, 23, 28]. Gene expression is readout of both the genetic and the environmental factors that contribute to the pathophysiology of schizophrenia. Analysis of human postmortem brain is a powerful approach for the identification of risk factors for schizophrenia, because unlike studies of living patients, detailed molecular investigations can be performed directly in the critical brain regions of interest.

The pathophysiology of schizophrenia is likely to be different between males and females. Sex differences have been noted in several epidemiological analyses. For example, several studies indicate that men have a slightly higher incidence of schizophrenia compared with women. In addition, males have an earlier age of onset of schizophrenia, between 18–25 years of age, compared with the female age of onset which is 25–35 years [20]. The symptoms exhibited by male and female patients with schizophrenia also differ. Males tend to have a greater vulnerability to negative symptoms and traits of disorganization, while females more frequently exhibit depressive symptoms [20]. These findings suggest that different underlying mechanisms of schizophrenia occur in males and females. Therefore, we have investigated sex differences in schizophrenia to gain a better understanding of the pathophysiological mechanisms underpinning this disorder.

To identify the biological factors involved in the pathogenesis of schizophrenia and how they are differentially influenced in the sexes, we have investigated microarray expression data from the prefrontal cortex (PFC) in postmortem brain. The PFC region has been strongly associated with deficits of executive function and other cognitive symptoms that occur in patients with schizophrenia. Gene expression within the PFC has been studied extensively using the microarray approach [4-6, 8, 9, 11, 13, 14, 16, 19, 24]. However, statistical analyses of gene expression data from individual small cohorts have lacked sufficient statistical power to avoid conflicting data in these different studies [17, 22]. Meta-analysis is a strategy by which these problems could be addressed, because the data from multiple studies can be combined, thus increasing the statistical power available. In this study, we have collected gene expression data generated from post-mortem cohorts of schizophrenia cases and psychiatrically healthy comparison groups that are included in publicly available databases. We have tested for sex differences in PFC gene expression in schizophrenia, using the meta-analysis paradigm.

## Methods and materials

### Public microarray datasets of postmortem gene expression in schizophrenia

We searched the public database and literature on the study conducted on PFC region of postmortem brains and decided to use Mistry’s merged expression dataset [17] for further analysis because this combined cohort contains the largest number of samples that could be accessed from available resources. In Mistry’s study, the raw image data of 306 postmortem brain samples from seven different datasets were first pooled together, Robust Multi-array Average (RMA) normalization procedure was then applied on these pooled samples to obtain normalized expression value of each probe set. Out of 306 samples, 246 are available to the public. The RMA normalized expression data (http://www.chibi.ubc.ca/wp-content/uploads/2013/02/combined.data.txt) and corresponding clinical data (http://www.chibi.ubc.ca/wp-content/uploads/2013/02/combined.design.txt) of these 246 samples are available for download on their website and will serve as a starting point in this study. The source of six studies available to the public in Mistry’s dataset is summarized in Table 1. ComBat [12] batch effect adjustment was carried out in R environment. We used ComBat() function included in the “sva” package downloaded from BioConductor website (https://www.bioconductor.org/packages/release/bioc/html/sva.html) to perform batch effect correction on the original dataset. Each study is treated as a batch and default parameter setup is used in running the ComBat function.

**Table 1:** Public postmortem microarray datasets used in this study

Data set	Brain region	Control: Schizophrenia
Stanley Bahn [35]	Frontal BA46	31(24M7F): 34(25M9F)
Stanley AltarC [35]	Frontal BA46/10	11(7M4F): 9(8M1F)
Mclean Harvard Brain Bank [36]	Prefrontal Cortex (BA9)	26(18M8F): 19(13M6F)
Mirnics [37]	Prefrontal Cortex (BA46)	6(4M2F): 9(5M4F)
Maycox GSE17612 [16]	Anterior prefrontal cortex (BA10)	21(12M9F): 26(18M8F)
Narayan GSE21138 [19]	Frontal (BA46)	29(24M5F): 25(21M4F)

### Differential expression analysis of each probe set

Expression values of each probe set were modeled using a fixed effect linear model approach, where disease status and imbalanced covariates between two groups are treated as fixed effects to be estimated from data. A model selection procedure was also employed for each probe set to address the confounding effect of imbalanced covariates. The details of the procedure will be described in next section. For each probe set, the t-statistic for the disease effect was then extracted from the selected model. P-values were computed using two-sided t-test. The resulting P-values were further converted to q-values using the *qvalue* package in R [25] which were defined as the minimum positive False Discovery Rate (q-value(t): Pr(H = 0|T > =t) in Bayesian interpretation over a nested rejection region containing observed statistics t) for multiple testing correction.

### Covariate adjustment

The observed covariate imbalance between two groups was analyzed to avoid generating misleading result. However modelling covariates for every gene could unnecessarily diminish statistical power if the covariate does not influence the expression of the gene. In our study, we consider only modelling imbalanced covariates for each probe set. We first obtained a probe set (gene) list where the covariate influence on the gene expression is determined with confidence. This refined gene list was generated using a previous method in a postmortem brain gene expression study, in which a correlation analysis was used to evaluate covariates such as age, post-mortem interval, brain PH etc. that influenced the expression of specific genes across multiple post-mortem normal brain datasets [18]. We extracted genes with meta-Q ≤ 0.01 to indicate that the gene was significantly influenced by a particular covariate, and separate it into two lists: positively-correlated and negatively-correlated. Notice that one probe set could be mapped to multiple genes, we excluded those probe sets appearing in the two lists. For each predefined covariate influenced probe set, we first modelled the expression value with a linear model including that covariate. If the direction of the fitted covariate estimates was inconsistent with the pre-refined list, we exclude this covariate and re-estimate a reduced model for this probe set. For the rest of the probe sets, no covariate adjustment was performed.

### Selection of sex-specific differentially expressed genes

To identify genes that had a sex by diagnosis interaction with schizophrenia, we used a strategy similar to [3]. Each individual in our dataset is assigned to one of four subgroups: Schizophrenia Male, Control Male, Schizophrenia Female and Control Female. Individuals could also be combined into two groups based on their diagnosis: Schizophrenia and Control group, or based on their sex: Male and Female group. Differential expression analysis was first performed using the procedure described in section 2.2 within each sex. After the initial probe set list was obtained from each sex, we further eliminated those probe sets that are associated with schizophrenia regardless of sex when all of the following criteria were met: (a) the difference between Schizophrenia and Control groups was statistically significant after multiple test correction (q-value < 0.05); (b) the fold change of Schizophrenia Female vs. Control Females, and Schizophrenia Male vs Control Males should be in the same direction i.e. both higher or both lower; (c) the expression difference was not significant between Male and Female groups (defined as *p* > 0.05 between Female and Male group). After removal of these probe sets from the initial probe set list, we sorted the remaining probe sets within each sex by q-value and report the top ranked probe sets (q-value < 0.05) as sex-specific differentially expressed genes.

### Function enrichment of differentially expressed genes

All differentially expressed genes, along with their Affymetrix ID numbers were imported into EASE (Expression Analysis Systematic Explorer) in DAVID (Database for Annotation, Visualization and Integrated Discovery), and were used to identify functionally significant gene classes (https://david.ncifcrf.gov/) [10]. This webserver uses statistical methods to map and identify functional gene categories (for example, Gene Ontology (GO), Kyoto Encyclopedia of Genes and Genomes (KEGG) or BioCarta), which are enriched in the significant gene list compared with their presence on the array.

## Results

### Batch and covariate adjustment

A total of 22277 probe sets is analyzed in this study. Before further analysis, by using hierarchical clustering analysis and Principal Component Analysis (PCA), we found it was necessary to correct for “batch effect” as samples from the same study were clustered together. ComBat [12] was used to correct this technical bias by treating each study as a batch. After ComBat adjustment, the hierarchical clustering and PCA results show that no significant clustering remained in the dataset and the adjusted expression data was suitable for further analysis (Additional file 1).

Clinical variables associated with each patient sample were examined to determine possible confounding variables. The age and postmortem interval (PMI) is well matched between schizophrenia and control groups while brain pH shows a significant difference between two groups (Table 2). Within each sex group, we observe that the brain PH is significantly different between schizophrenia males and control males, on the other hand, all covariates are well balanced in female group (Table 2). Age at death was significantly different between male and female group (Additional file 2). In the predefined brain PH list, 2413 probe sets’ expressions were positively correlated with the covariate and 893 were negatively correlated; in the predefined age related list, the number is 1907 and 3028 respectively. The imbalanced covariates and the proportion of probe sets subject to covariate adjustment in each differential analysis are summarized in Table 3.

**Table 2:** Demographic data of postmortem subjects

	Total sample			Male group			Female group		
	Control	SCZ	p-val	Control	SCZ	p-val	Control	SCZ	p-val
Size	124	122		89	90		35	32	
Age	50.29 ± 17.2	51.09 ± 18.90	0.73	47.85 ± 16.44	48.45 ± 17.71	0.81	56.49 ± 17.89	58.53 ± 20.38	0.67
Gender	89 M:35 F	90 M:32 F	>0.05	NA	NA	NA	NA	NA	NA
Brain PH	6.48 ± 0.29	6.36 ± 0.29	0.002	6.50 ± 0.29	6.35 ± 0.26	<0.001	6.42 ± 0.29	6.40 ± 0.38	0.78
PMI^3^	25.49 ± 14.8	25.09 ± 15.9	0.84	26.12 ± 14.37	25.79 ± 16.45	0.89	23.91 ± 15.98	23.14 ± 14.34	0.84

*Abbreviations*: *SCZ* Schizophrenia, *p-val* p-value, *M* Male, *F* Female, *PMI* post-mortem interval

The summary demographics (mean ± s.d.) and t-test P-values for group difference are shown. For sex difference, we report the P-value generated from a chi-square test for equality of proportions

**Table 3:** Covariate adjustment summary

Two group comparison	Schizophrenia vs control	Schizophrenia-male vs control-male	Male vs Female
Imbalanced covariate between two groups	Brain PH	Brain PH	Age
Proportion of probe sets adjusted^a^ (%)	11.87 %	11.47 %	18.00 %

^a^A total of 22777 probe sets is analyzed in this study

### Genes with altered PFC expression in schizophrenia

**Fig. 1:**
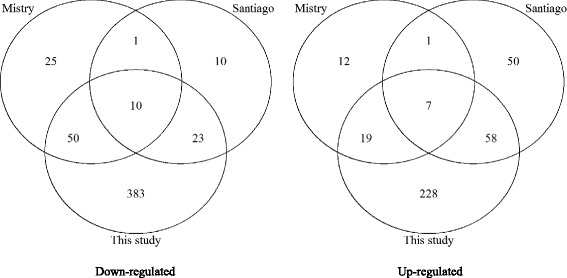
Comparison of meta-analysis results among three studies

To validate our analytical approach used in this study, we performed differential analysis between Schizophrenia and Control group and compared the derived gene list with two published results in which similar meta-analysis were performed [17, 22]. We identified 466 probe sets (representing 427 unique genes) that were significantly down-regulated in the schizophrenia cases relative to the controls and 312 probe sets (representing 261 unique genes) significantly up-regulated in schizophrenia with q-value < 0.05. Our results show that a large number of overlapped probe sets were observed between our gene list and the other two studies (Fig. 1). All overlapped probe sets showed the same direction of fold difference between the schizophrenia cases and controls. In comparison with result of Mistry et al. [27], 86 out of 125 probe sets (68.8 %) were also identified by our approach. In comparison with result of Santiego et al., 98 out of 160 probe sets (61.3 %) overlapped with our result [22]. Our method identified a similar proportion of the probe sets from both studies.

We also examined whether the identified 778 probe sets are associated with schizophrenia in case control studies of genetic polymorphisms. We compared our gene list with those deposited in SZGene (www.SZgene.org) database which contains the most comprehensive review of schizophrenia association studies [31]. 80 probe sets representing 68 unique genes are found to be genetically associated with schizophrenia. Genes previously showing strong genetic evidence implicated in schizophrenia identified in this study include: regulator of G-protein signaling 4 (RGS4) [32]; discoidin domain receptor family, member 1(DDR1) [33]; and the selenium binding protein 1(SELENBP1) [34]. The full result is included in Additional file 3.

### Sex differences in PFC gene expression in schizophrenia

In the male group, we first identified 138 differentially expressed probe sets with a q-value <0.05. We then removed 80 probe sets which shows differential expression regardless of sex based on the filter defined in the Method part. 50 probe sets representing 46 unique genes were identified as specifically different in male schizophrenia patients relative to male controls: 23 probe sets had lower expression (Table 4) and 27 probe sets have higher expression (Table 5) in males with schizophrenia. In the female group, we were not able to identify any differentially expressed probe sets with q-value <0.05 after multiple testing correction.

**Table 4:** Genes with altered expression in males with schizophrenia: *downregulated probe sets*

Probe set	Gene symbol	Locus	Description	Fold difference	q-value
209735_at	ABCG2	4q22	ATP-binding cassette, sub-family G (WHITE), member 2	−1.29	0.031
208868_s_at	GABARAPL1	12p13.2	GABA(A) receptor-associated protein like 1	−1.17	0.043
208813_at	GOT1	19q24.1-q25.1	glutamic-oxaloacetic transaminase 1, soluble (aspartate aminotransferase 1)	−1.17	0.043
212878_s_at	KLC1	14q32.3	kinesin light chain 1	−1.14	0.044
208002_s_at	ACOT7	1p36	acyl-CoA thioesterase 7	−1.14	0.033
213897_s_at	MRPL23	11p15.5	mitochondrial ribosomal protein L23	−1.10	0.043
211382_s_at	TACC2	10q26	transforming, acidic coiled-coil containing protein 2	−1.10	0.033
214365_at	TPM3	1q21.2	tropomyosin 3	−1.10	0.037
213738_s_at	ATP5A1	18q21	ATP synthase, H+ transporting, mitochondrial F1 complex, alpha subunit 1, cardiac muscle	−1.10	0.031
221909_at	RNFT2	12q24.22	ring finger protein, transmembrane 2	−1.10	0.033
201322_at	ATP5B	12q13.13	ATP synthase, H+ transporting, mitochondrial F1 complex, beta polypeptide	−1.10	0.049
203272_s_at	TUSC2	3p21.3	tumor suppressor candidate 2	−1.09	0.049
218332_at	BEX1	Xq22.1	brain expressed, X-linked 1	−1.09	0.033
201077_s_at	NHP2L1	NA	NHP2 non-histone chromosome protein 2-like 1 (S. cerevisiae)	−1.09	0.041
201410_at	PLEKHB2	2q21.1	pleckstrin homology domain containing, family B (evectins) member 2	−1.08	0.033
219760_at	LIN7B	19q13.3	lin-7 homolog B (C. elegans)	−1.08	0.049
221315_s_at	FGF22	19p13.3	fibroblast growth factor 22	−1.08	0.042
221706_s_at	USE1	19p13.11	unconventional SNARE in the ER 1 homolog (S. cerevisiae)	−1.07	0.042
202967_at	GSTA4	6p12.1	glutathione S-transferase alpha 4	−1.07	0.049
207839_s_at	TMEM8B	9p13.3	transmembrane protein 8B	−1.07	0.042
221746_at	UBL4A	Xq28	ubiquitin-like 4A	−1.07	0.033
208971_at	UROD	1p34	uroporphyrinogen decarboxylase	−1.07	0.043
202486_at	AFG3L2	18p11	AFG3 ATPase family gene 3-like 2 (S. cerevisiae)	−1.06	0.044

**Table 5:** Genes with altered expression in males with schizophrenia: *upregulated probe sets*

Probe set	Gene symbol	Locus	Description	Fold difference	q-value
212226_s_at	PPAP2B	1p32.2	phosphatidic acid phosphatase type 2B	1.32	0.043
202975_s_at	RHOBTB3	5q15	Rho-related BTB domain containing 3	1.28	0.031
202935_s_at	SOX9	17q24.3	SRY (sex determining region Y)-box 9	1.28	0.043
202887_s_at	DDIT4	10q22.1	DNA-damage-inducible transcript 4	1.26	0.041
212859_x_at	MT1E	16q13	metallothionein 1E	1.26	0.031
200897_s_at	PALLD	4q32.3	palladin, cytoskeletal associated protein	1.24	0.031
200907_s_at	PALLD	4q32.3	palladin, cytoskeletal associated protein	1.23	0.033
209210_s_at	FERMT2	14q22.1	fermitin family member 2	1.22	0.033
213016_at	BBX	3q13.1	bobby sox homolog (Drosophila)	1.21	0.031
213158_at	unknown	3q13.2	unknown	1.21	0.042
201029_s_at	CD99	Xp22.32/Yp11.3	CD99 molecule	1.18	0.043
218350_s_at	GMNN	6p22.3	geminin, DNA replication inhibitor	1.17	0.033
214212_x_at	FERMT2	14q22.1	fermitin family member 2	1.16	0.033
209069_s_at	H3F3B	NA	H3 histone, family 3B (H3.3B)	1.16	0.037
205475_at	SCRG1	4q34.1	stimulator of chondrogenesis 1	1.15	0.041
208022_s_at	CDC14B	9q22.3	CDC14 cell division cycle 14 homolog B (S. cerevisiae)	1.14	0.039
211997_x_at	H3F3B	NA	H3 histone, family 3B (H3.3B)	1.14	0.038
41644_at	SASH1	6q24.3	SAM and SH3 domain containing 1	1.13	0.044
215811_at	Unknown gene	NA	-	1.13	0.047
209600_s_at	ACOX1	17q25.1	acyl-CoA oxidase 1, palmitoyl	1.13	0.033
202771_at	FAM38A	16q24.3	family with sequence similarity 38, member A	1.11	0.042
203636_at	MID1	Xp22	midline 1 (Opitz/BBB syndrome)	1.11	0.049
200906_s_at	PALLD	4q32.3	palladin, cytoskeletal associated protein	1.10	0.042
213342_at	YAP1	11q13	Yes-associated protein 1	1.09	0.031
210105_s_at	FYN	6q21	FYN oncogene related to SRC, FGR, YES	1.09	0.033
215823_x_at	PABPC1 /// RLIM	NA	poly(A) binding protein, cytoplasmic 1 /// ring finger protein, LIM domain interacting	1.08	0.044
210094_s_at	PARD3	10p11.21	par-3 partitioning defective 3 homolog (C. elegans)	1.08	0.041

### Function annotation of male-specific differentially expressed genes

The functions of 46 genes associated with schizophrenia in male patients were manually inspected. Of the genes with significantly lower expression in males with schizophrenia, several were related to energy metabolism (ATP5B, ATP5A1, MRPL23, AFG3L2, ABCG2). 4 genes (BEX1, UBL4A, CD99 and MID1) located on the sex chromosome are identified. We also detected differential expression of 4 genes in the males with schizophrenia that were previously identified by Mistry et al. [17] in which sex was not considered. These were Rho-Related BTB Domain-Containing Protein 3 (RHOBTB3), Bobby Sox homolog (BBX), H3 Histone, Family 3B (H3F3B) and pleckstrin homology domain containing, family B (evectins) member 2 (PLEKHB2). Finally, using DAVID webserver, we performed an enrichment analysis to systematically identify over-representation of biological processes or pathways that are altered in the PFC in male schizophrenia patients. After correction for multiple comparisons, we were unable to identify any significant biological process (False Discovery Rate <0.05) in the GO term Biological Process database. We achieved similarly negative results using the KEGG pathway database.

## Discussion

We have reported the gene expression differences that show a sex by diagnosis interaction in the PFC in schizophrenia. To our knowledge, this is the first study using a meta-analytical approach to identify sex differences in this brain region in patients with schizophrenia. There are limited data on sex differences in schizophrenia at a molecular level [15, 29], although evidence from epidemiological and animal studies indicates that sex differences exist in this disorder [20, 27, 30]. Individual post-mortem gene expression studies have low statistical power to identify gene expression differences in schizophrenia. This is, most often due to the small sample sizes and moderate gene expression differences between the diagnostic groups. Meta-analysis, on the other hand, addresses this problem and increases statistical power by combining samples from different subject cohorts. The results obtained from our meta-analysis are robust at the statistical level [17]. Our findings open a new window to understand the different pathophysiological mechanisms that lead to schizophrenia in males and females.

In the differential analyses between schizophrenia and control group, we identified the most number of genes most of which overlapped with the results of two previously published studies, while the other two published studies had little overlap between each other (Fig. 1). To find the reason for the difference, we run the same procedure as in Mistry’s for our dataset. We find that the main difference comes from different treatment of “batch effect”. In Mistry’s study, they treated each experiment date and study as batch variable. A total of 50 batch date and 6 study was modelled in their linear model framework. Introduction of too many predictor variables will decrease the degrees of freedom of t-test on the estimated disease effect coefficient, thus decreasing the likelihood of rejecting the null hypothesis, leading to higher Type-II error. Based on the observation of hierarchical clustering and PCA result, we consider that modelling each study alone would be sufficient to correct the batch effect. The second reason explaining the difference is covariate adjustment. We only include a covariate in the model when its influence on the gene is confirmed with confidence; while in Mistry’s study, they assumed that the covariate influenced every gene resulting in unnecessary inclusion of unrelated variables in the model. Therefore the approach taken in this study has more detection power and leads to discovery of more genes.

Our analyses identified 46 genes that were differentially expressed specifically in male patients with schizophrenia. This finding of 50 probe sets is much larger than the expected number of false positives according to our selection procedure. The expected number of false positives was calculated to be 6.9 (138*0.05 = 6.9). Five genes were related to energy metabolism (ATP5B, ATP5A1, MRPL23, AFG3L2, ABCG2). Genes from this function category are consistently implicated in studies of schizophrenia. Another gene that had altered expression in the male schizophrenia group encodes γ-aminobutyric acid receptor-associated protein-like 1 (GABARAPL1) is an early estrogen-induced gene that when overexpressed, interacts with GABA-A or κ-opioid receptors, and plays a role in cell proliferation and cellular metabolic processes [1]. Function enrichment analysis generates negative results for these genes, as indicated in other microarray studies of the PFC in schizophrenia [17]. These results suggest that a diverse number of molecular functions are disrupted in males with schizophrenia.

No genes could be identified in female group after multiple testing correction (q-value < 0.05). To determine if this is due to a much smaller sample size than in male group, we randomly picked the same number of control and schizophrenia subjects from the male groups ten times and run differential analysis on these samples. The procedure was repeated 100 times. No significant difference could be detected in the expression level of any gene in the phenotype groups (data not shown). We then gradually increase the number of samples until genes could be detected with q-value < 0.05 in each run. We started to identify differentially expressed genes when there are 60 controls and 60 schizophrenia in the sample pool. This analysis showed that increasing the number of samples would improve the likelihood of identifying schizophrenia-associated genes in the females.

Very few studies have been conducted of sex differences in gene expression in the PFC in schizophrenia. Vawter et.al. [29] reported that three genes (MDH1, HINT1 and SERPINI1) had decreased expression in PFC region of 13 male schizophrenia patients compared with 11 male controls and no expression difference was observed in comparing 9 female schizophrenia patients with 10 female controls using quantitative PCR. We then extracted the corresponding probe sets from our dataset and summarized the result in Table 6. We observed significantly lower expression levels of all three genes in the schizophrenia group compared with controls (Table 6, Column 3 and 4). The expression difference of these 3 genes in was also tested in males and females separately (Table 6, Column 5 to 8). Our analysis suggests that all three genes might be altered by schizophrenia and are not related to sex difference. For MDH1, the expression is significantly decreased in the schizophrenia group of both sexes suggesting that this gene might be down-regulated in schizophrenia regardless of sex. HINT1 and SERPINI1 do not show differential expression between the male and female groups (Table 6, Column 9). Our study has a larger sample size than that of Vawter et al. thus we have greater statistical power to detect small effects, and we would argue that these genes are associated with schizophrenia but are not differentially expressed between the sexes.

**Table 6:** Genes reported by Vawter et al. [29] in this study

		Total sample		Male group		Female group		Sex difference^a^
Probe set	Gene symbol	Fold change	Q-value	*P*-value	Fold change	P-value	Fold change	*P*-value
200978_at	MDH1	−1.11	0.007	0.000491	−1.10	0.009	−1.14	0.061
207721_x_at	HINT1	−1.08	0.014	0.00051	−1.08	0.065	−1.08	0.062
205352_at	SERPINI1	−1.10	0.027	0.010158	−1.08	0.091	−1.16	0.073

^a^Two group t-test comparison between male and female group

## Conclusion

In summary, this is the most comprehensive and up-to-date analysis of sex differences in prefrontal cortex gene expression in schizophrenia. Some of our data consolidate the reports of previously published papers. Our results indicate that further investigation of sex differences in schizophrenia is required [15]. These data bring us closer to understanding the different molecular mechanisms underpinning schizophrenia in males and females, so that novel targets for antipsychotic drug development can be identified. Genes with altered expression in schizophrenia can also serve as biological markers for the disorder, so that biochemical diagnostic tools can facilitate the practice of clinical psychiatry.

## Additional files

Additional file 1: Figure S1. Comparisons of hierarchical clustering and PCA results before and after ComBat adjustment. (DOCX 665 kb) Additional file 2: Table S1. Demographic comparison between male and female group. (XLSX 9 kb) Additional file 3: Table S2. 778 schizophrenia-affected probe sets identified in this study. (XLSX 66 kb)
