# Supplementary material for: Meta-analysis of sex differences in gene expression in schizophrenia
Source: BMC Syst Biol. 2016 Jan 11;10(Suppl 1):9. doi: 10.1186/s12918-015-0250-3 (PMC4895727; doi:10.1186/s12918-015-0250-3)

Supplementary Figure: Comparisons of hierarchical clustering and PCA results before and after ComBat adjustment.

A. Hierarchical clustering results before ComBat adjustment:

B. Hierarchical clustering results after ComBat adjustment:

C. PCA results before ComBat adjustment:


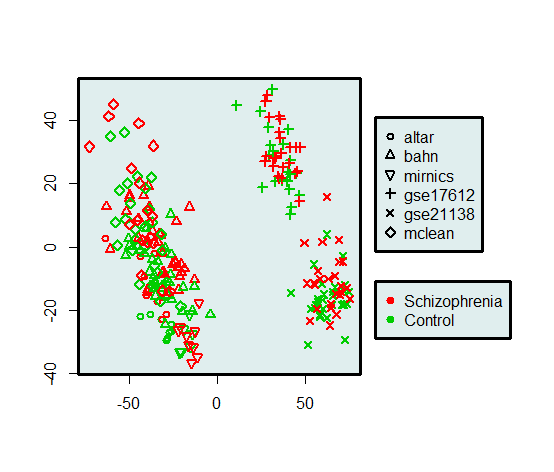


D. PCA results after ComBat Adjustment:


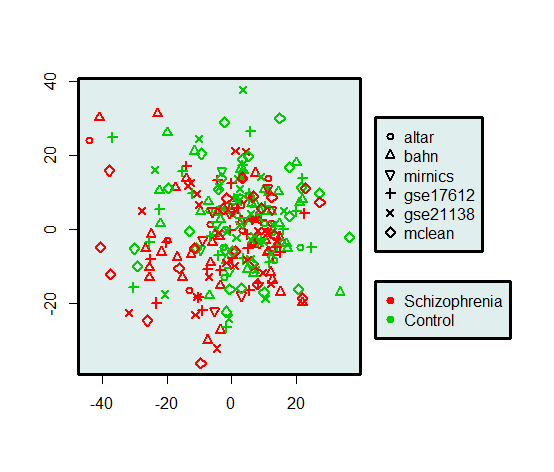

Supplement: Additional file 1: Figure S1. — Comparisons of hierarchical clustering and PCA results before and after ComBat adjustment. (DOCX 665 kb) [file 12918_2015_250_MOESM1_ESM.docx]
